# The separate axes of TECPR1 and ATG16L1 in CASM

**DOI:** 10.1080/15548627.2023.2255462

**Published:** 2023-09-07

**Authors:** Namrita Kaur, Sven R Carlsson, Alf Håkon Lystad

**Affiliations:** a Centre for Cancer Cell Reprogramming, Faculty of Medicine, University of Oslo, Oslo, Norway; b Department of Molecular Cell Biology, Institute for Cancer Research, Oslo University Hospital, Oslo, Norway; c Department of Medical Biochemistry and Biophysics, University of Umeå, Sweden

**Keywords:** CASM, DysF, membrane damage, non-canonical autophagy, SopF, sphingomyelin

## Abstract

Conjugation of ATG8 to single membranes (CASM) is a fundamental cellular process that entails the conjugation of mammalian Atg8 homologs, here referred to as ATG8, to phosphatidylethanolamine (PE) and phosphatidylserine (PS) on endolysosomal compartments. Our current research, together with recent reports from the Randow, Wu, and Wileman labs, has uncovered yet another layer to this process. We discovered that, in addition to ATG16L1-containing complexes, TECPR1 (tectonin beta-propeller repeat containing 1)-containing ATG12–ATG5 E3 complexes can facilitate CASM, thereby providing a broader understanding of this pathway.

ATG16L1, previously considered essential for CASM, associates with the V-ATPase in response to shifts in endolysosomal proton gradients. This connection forms the ATG16L1-V-ATPase axis (Figure 1), a specialized cascade of the CASM process. This axis allows ATG16L1 to facilitate CASM when there is a dissipation in the proton gradients within the endolysosomal compartments.

**Figure 1.**
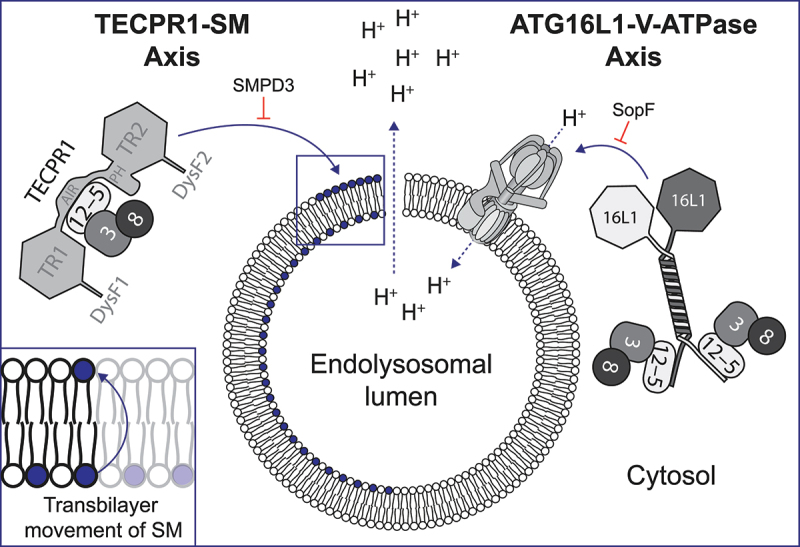
Illustration of the TECPR1-SM and ATG16L1-V-ATPase axes in CASM. The figure illustrates endolysosomal membrane damage, leading to proton gradient dissipation and sphingomyelin (SM) exposure on the cytosol-facing leaflet of the membrane, with an inset showing the transbilayer movement of SM. On the left, ATG12–ATG5 (12–5)-TECPR1 and ATG3 (3) conjugated to an Atg8-family protein (8) are recruited to exposed SM. On the right, the ATG12–ATG5-ATG16L1 (16L1) complex with the ATG3–Atg8-family protein is recruited by the V-ATPase. SMPD3/nSMase2 overexpression, converting SM to ceramide, inhibits TECPR1 recruitment (left), while SopF blocks recruitment of ATG16L1 (right). The key domains of TECPR1 are highlighted: tectonin propellers (TR), pleckstrin homology domain (PH), ATG5-interacting region (AIR) and DYSF domains (DysF).

In a new development, our research reveals that TECPR1 forms an active E3 complex in CASM, responding to endolysosomal membrane damage [1]. Specifically, when membrane damage occurs, it leads to a distress signal manifested as the cytosolic exposure of sphingomyelin (SM). This, in turn, recruits TECPR1 to the damaged areas via its DysF domains (DysF1 and DysF2), resulting in ATG8 lipidation. The DysF domains bind directly to SM and are crucial to the recruitment of TECPR1 as well as its E3-like function during ATG8ylation. Importantly, overexpressing SMPD3/nSMase2, an enzyme that converts SM to ceramide, abolishes TECPR1 activity and recruitment. This finding establishes the TECPR1-SM axis as a new functional unit in CASM (Figure 1).

DysF domains are not exclusive to TECPR1; they are also found in MYOF (myoferlin) and DYSF (dysferlin). However, there is a significant difference – in these proteins, one DysF domain is inserted into another, forming a unique extended structure. Intriguingly, as with TECPR1, both MYOF and DYSF respond to membrane damage, suggesting a potentially broader role for DysF domains in cellular responses to membrane damage.

TECPR1 also contains a membrane-binding pleckstrin homology (PH) domain that can greatly influence its E3 activity. Located adjacent to the ATG5 binding site, also known as the ATG5-interacting region (AIR), we propose that the PH domain plays an instrumental role in ensuring the precise orientation and proximity to lipids necessary for the conjugation reaction to work. Thus, the PH domain could then function similarly to helix 2 in ATG16L1; however, this remains a subject for more detailed investigation.

One of our intriguing findings is that TECPR1 continues to maintain its E3 functionality even in the presence of SopF, a virulence factor from the bacterium *Salmonella* Typhimurium, renowned for blocking ATG16L1-mediated CASM. This leads us to a broader understanding of CASM, uncovering the cooperative interplay between ATG16L1 and TECPR1. While ATG16L1 is critical in certain conditions, TECPR1 becomes prominent when ATG16L1’s function is impaired. Particularly in scenarios involving SopF, the collaboration of ATG16L1 and TECPR1 provides an evolutionary advantage, suggesting the possibility of a dual mechanism that may also prove advantageous in other contexts.

In conclusion, TECPR1 is essential in CASM, initiating the conjugation of ATG8 to PS and PE when SM is exposed to the cytosol. Thus, TECPR1 provides a complementary mechanism to that of ATG16L1, ensuring the continuation of CASM under various conditions. The most important difference between the two CASM axes lies in the mechanism by which the endolysosomal damage is detected. Whereas ATG16L1 relies on detecting changes in the pH gradient, TECPR1 is designed to detect a disturbance in the asymmetry of membrane lipids. It appears that different agents (chemicals and pathogens) can affect these membrane processes differently. Therefore, in order to have a broader repertoire of damage detection and a more effective and stronger defense, evolution has developed several alternative pathways to induce CASM.
